# Rewiring neural circuits by the insertion of ectopic electrical synapses in transgenic *C. elegans*

**DOI:** 10.1038/ncomms5442

**Published:** 2014-07-16

**Authors:** Ithai Rabinowitch, Marios Chatzigeorgiou, Buyun Zhao, Millet Treinin, William R. Schafer

**Affiliations:** 1 Division of Cell Biology, MRC Laboratory of Molecular Biology, Francis Crick Avenue, Cambridge CB2 0QH, UK; 2 Department of Medical Neurobiology, Hadassah Medical School, Hebrew University of Jerusalem, Jerusalem 9112102, Israel; 3 These authors contributed equally to this work

## Abstract

Neural circuits are functional ensembles of neurons that are selectively interconnected by chemical or electrical synapses. Here we describe a synthetic biology approach to the study of neural circuits, whereby new electrical synapses can be introduced in novel sites in the neuronal circuitry to reprogram behaviour. We added electrical synapses composed of the vertebrate gap junction protein Cx36 between *Caenorhabditis elegans* chemosensory neurons with opposite intrinsic responses to salt. Connecting these neurons by an ectopic electrical synapse led to a loss of lateral asymmetry and altered chemotaxis behaviour. In a second example, introducing Cx36 into an inhibitory chemical synapse between an olfactory receptor neuron and an interneuron changed the sign of the connection from negative to positive, and abolished the animal’s behavioural response to benzaldehyde. These data demonstrate a synthetic strategy to rewire behavioural circuits by engineering synaptic connectivity in *C. elegans*.

## Abstract (web summary)

Neural circuits are functional ensembles of neurons that are selectively interconnected by chemical or electrical synapses. Here the authors describe an approach to the study of neural circuits in *C. elegans* whereby electrical synapses are introduced between previously unconnected neurons to reprogram behaviour.

## Graphical abstract

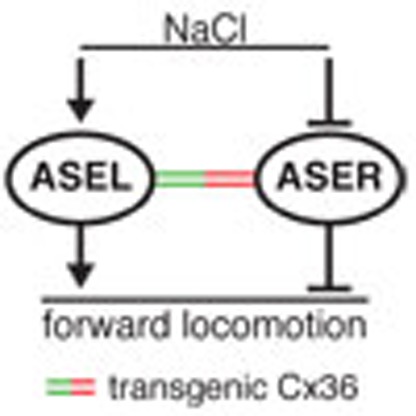

A central aim of neuroscience research is to reveal the function of neural circuits and how changes in synaptic connectivity modify behaviour. Recent advances in synthetic biology, whereby genetic networks of living cells have been rewired to produce alternative cellular function^1^,^2^,^3^, may revolutionize the field of cell biology. Synthetic approaches for re-engineering neural function and behaviour have also begun to be developed for neuroscience; in particular, optogenetic and other protein-based reagents have been used to manipulate the activity patterns of defined neurons *in vivo*^4^,^5^. In principle, a synthetic neurobiology approach could also be used to manipulate neural circuits by artificially modifying specific synaptic connections in the circuit and thus reprogramming behaviour.

To artificially modify the connection between neurons we sought to introduce a new synapse into an existing neural circuit. Inserting heterologous chemical synapses into a circuit would be difficult owing to their enormous complexity and hundreds of constituent proteins^6^,^7^. In contrast, electrical synapses, or gap junctions, consist of as little as one protein type^8^, and it has been shown that overexpression of an endogenous gap junction protein can alter neuronal connectivity *in vitro*^9^. The molecular constituents of vertebrate and invertebrate gap junctions belong to distinct protein families: connexins are exclusive to vertebrates and innexins to invertebrates^10^,^11^,^12^. Importantly, while proteins from the same family can interact to form heterotypic gap junctions^13^,^14^, no compatibility has been found between connexins and innexins^15^. Thus, we reasoned that connexins expressed heterologously in *Caenorhabditis elegans* neurons would form gap junctions exclusively between themselves, and that these new connections could be used to introduce novel connections between normally unconnected neurons in intact animals. Here we present data assessing the use of this approach in two neural circuits, involved in salt taste and olfactory chemotaxis.

## Results

### Adding synthetic electrical synapses between sensory neurons

**Figure 1:**
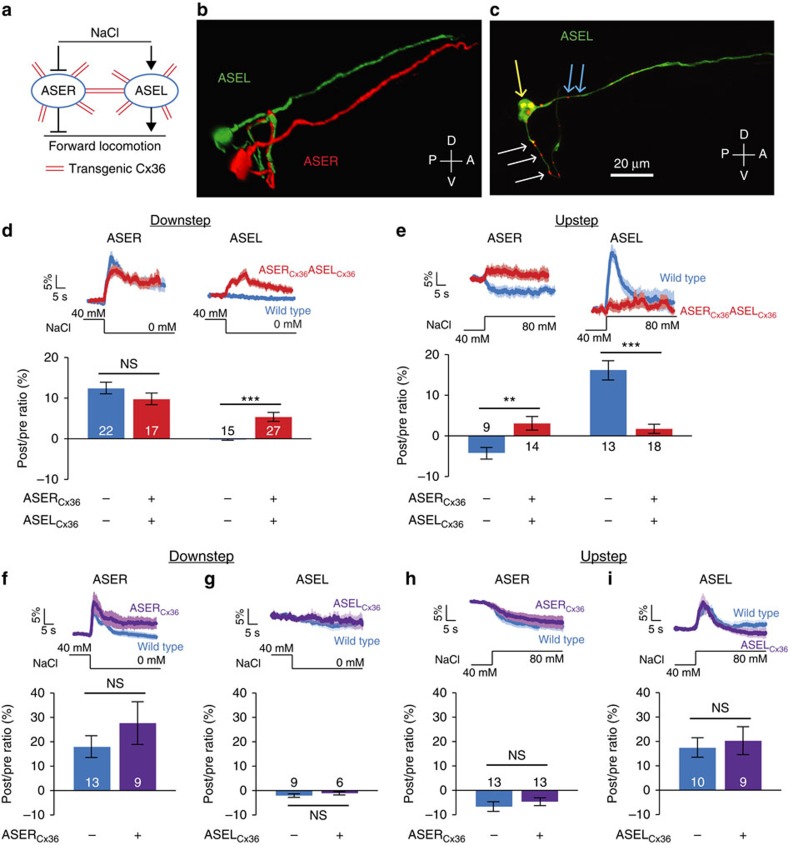
An engineered electrical synaptic connection functionally couples ASER and ASEL. (**a**) ASEL and ASER participate in a circuit for salt sensation and do not have any natural electrical connections. Chemical synapses between ASEL and ASER may be present in wild-type animals^22^, although the loss of synaptic transmission is not reported to affect ASEL and ASER sensory responses^18^. (**b**) The axons of ASER (red) and ASEL (green) appear to contact each other, as revealed by confocal microscopy. Shown is a three-dimensional confocal image of strain AQ3309 (*gcy-7::gfp, gcy-5::mCherry*; see strain list). (**c**) Confocal image of strain AQ3311, in which ASEL (green) expresses the genetically encoded calcium indicator YC3.60 and Cx36 tagged with mCherry (red). Cx36 puncta in the cell body (yellow arrow), dendrite (blue arrows) and axon (white arrows) are indicated. Scale bar, 20 μm. (**d**,**e**) Calcium imaging of ASER and ASEL responses to a downstep (**d**) or upstep (**e**) in NaCl concentration in wild-type worms and in worms expressing Cx36 in both ASER and ASEL. (**f**–**i**) Calcium imaging of ASER (**f**,**h**) or ASEL (**g**,**i**) responses to a downstep (**f**,**g**) or upstep (**h**,**i**) in NaCl concentration in wild-type worms and in worms expressing Cx36 in ASER (**f**,**h**) or ASEL (**g**,**i**) alone. At the top of each panel are averaged traces with shaded regions indicating s.e.m.; blue traces indicate wild-type, purple traces single neuron connexin lines, and red traces lines expressing Cx36 in both ASEL and ASER. At the bottom of each panel are percent mean fluorescent ratios, 10 s after compared with 10 s before stimulus onset. Two-tailed unpaired *t*-tests, **P<0.01, ***P<0.001, NS, not significant. Error bars represent s.e.m. Numbers on bars indicate sample sizes.

We first investigated the possibility of rewiring neuronal connections *in vivo* using the neural circuit involved in salt chemotaxis. *C. elegans* navigates up gradients of sodium chloride and other salts in part through a biased random walk^16^, combining forward travel with stochastically occurring reorienting turnings and reversals. The primary neurons sensing attractive concentrations of salt are the bilaterally symmetric ASE neurons^17^; ASEL is an ON cell that is stimulated by an increase in salt concentration, while ASER is an OFF cell stimulated by a salt concentration decrease^18^. Unlike most bilateral sensory neuron pairs in *C. elegans*, the ASEL and ASER neurons are not normally connected by gap junctions (Fig. 1a). Published connectome data likewise report no chemical synapses between ASEL and ASER^19^,^20^, although more recent online data based on computer-aided reconstructions^21^,^22^ suggest some may exist. Moreover, confocal imaging of ASER and ASEL, labelled with expressed fluorescent proteins of different colours, showed that the processes of these two neurons are physically adjacent within the nerve ring (Fig. 1b).

We therefore reasoned that heterologous connexin expression could be used to synthetically connect the ASEL and ASER neurons with electrical synapses. We generated a synthetic connexin-derived transgene consisting of a *C. elegans* codon-optimized^23^, fluorescently tagged version of the mouse connexin 36 (Cx36), a well-characterized, broadly expressed gap junction constituent^24^, under the control of promoters specific for either ASER (*gcy-5*) or ASEL (*gcy-7*). In multicopy extrachromosomal lines overexpressing Cx36 in both neurons (ASER_Cx36_ASEL_Cx36_), as well as a line expressing Cx36 in ASER alone (ASER_Cx36_), this tagged Cx36 protein localized throughout the dendrite, axon and cell body in a punctate pattern (Fig. 1c). In some but not all cases, ASER_Cx36_ puncta were coincident to ASEL_Cx36_ puncta, suggesting that they might represent synthetic gap junctions (Supplementary Fig. 1).

To test whether Cx36 expression could indeed generate ectopic gap junctions linking ASEL and ASER, we performed calcium-imaging experiments to investigate the effects of ASER_Cx36_ and ASEL_Cx36_ on neuronal responses to chemosensory stimulation. Although genetically encoded calcium indicators lack the temporal sensitivity to detect rapid electrical potentials, they are well-suited to detect changes in the slow, graded responses to NaCl expected from the introduction of electrical coupling between ASEL and ASER. In wild-type animals, a step change from 40 to 0 mM NaCl evoked a calcium increase in ASER but no response in ASEL (Fig. 1d, blue). Expressing Cx36 in ASEL or ASER alone did not significantly alter either response (Fig. 1f,g). In contrast, in ASER_Cx36_ASEL_Cx36_ animals both ASEL and ASER showed robust calcium increases in response to the downstep stimulus (Fig. 1d, red), as predicted if the cells were electrically coupled. We also assayed the responses of Cx36-expressing strains to increases in NaCl concentration. We observed that in wild-type, ASEL_Cx36_ and ASER_Cx36_ animals, salt upsteps led to a calcium increase in ASEL and a calcium decrease in ASER (Fig. 1e,h,i). However, in ASER_Cx36_ASEL_Cx36_ animals (Fig. 1e, red), ASER showed a calcium increase in response to a salt upstep, as expected if the depolarizing current in ASEL depolarized ASER through an electrical connetion. These alterations in ASE activity correlated with a significant defect in the ability of the animals to navigate in a point-source gradient of NaCl (Supplementary Fig. 2a). Together, these results indicate that functional electrical synapses were successfully inserted between ASEL and ASER.

### Flipping the sign of an inhibitory synapse

**Figure 2:**
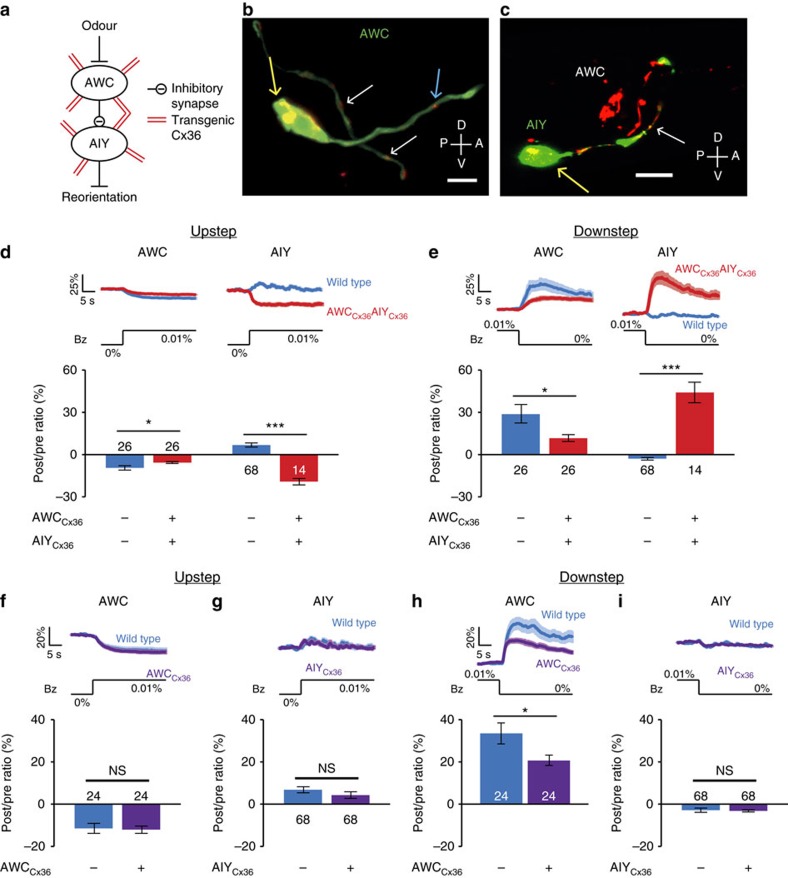
An engineered electrical synaptic connection between AWC and AIY flips the AIY benzaldehyde response profile. (**a**) AWC and AIY participate in a circuit for chemosensation and are naturally connected by an inhibitory chemical synapse. (**b**) Confocal projection image of strain AQ2660, in which AWC (green) expresses the calcium indicator YC3.60, and mCherry-tagged Cx36 puncta (red) are seen in the cell body (yellow arrow), dendrite (blue arrow) and axon (white arrows). Scale bar, 5 μm. (**c**) Confocal image of strain transgenic line AQ2646, in which mCherry-tagged Cx36 is expressed in AIY and AWC (red punctate fluorescence), and YC3.60 is expressed in AIY (green fluorescence). Yellow and white arrows indicate the cell body and axon respectively. Scale bar, 10 μm. An expanded version of this image and version showing the red channel alone are in Supplementary Fig. 3. (**d**,**e**) Calcium imaging of AWC and AIY responses to an upstep (**d**) or downstep (**e**) in benzaldehyde (Bz) concentration in wild-type worms and in worms expressing Cx36 in both AWC and AIY. (**f**–**i**) Calcium imaging of AWC (**f**,**h**) or AIY (**g**,**i**) responses to an upstep (**f**,**g**) or downstep (**h**,**i**) in Bz concentration in wild-type worms and in worms expressing Cx36 in AWC (**f**,**h**) or AIY (**g**,**i**) alone. At the top of each panel are averaged traces with shaded regions indicating s.e.m. At the bottom of each panel are percent mean fluorescent ratios, 10 s after compared with 10 s before stimulus onset. Two-tailed unpaired *t*-tests, *P<0.05, ***P<0.001, NS, not significant. Error bars represent s.e.m. Numbers on bars indicate sample sizes.

We next tested whether existing neuronal connections could be reconfigured through ectopic Cx36 expression. To do this we focused on the AWC and AIY neurons, which mediate chemotaxis towards attractive odourants, such as benzaldehyde^25^,^26^. The AWC sensory neurons respond to increases in odour concentration with reduced activity and to decreases in odour concentration with elevated activity. The AWCs make inhibitory chemical synapses with the AIY interneurons (Fig. 2a); although AIY ablation does not impair chemotaxis to odourants^27^, optogenetic AIY activation has been shown to promote forward travel and inhibit reorientations^28^. To determine whether heterologous connexin expression could modify the properties of these synapses and thereby impact behaviour, we expressed Cx36 in AWC and AIY using promoters derived from *odr-1* (for AWC_Cx36_) and the *ttx-3* second intron (for AIY_Cx36_) and imaged calcium responses to olfactory stimuli using genetically encoded calcium indicators (Fig. 2b,c; Supplementary Fig. 3). In wild-type animals, benzaldehyde presentation led to a calcium decrease in AWC and a reciprocal calcium increase in AIY, as expected from their known inhibitory connection (Fig. 2d). In animals expressing either the AWC_Cx36_ or the AIY_Cx36_ transgenes alone, these responses were not significantly altered (Fig. 2f,g). In contrast, animals expressing Cx36 in both neurons (AWC_Cx36_AIY_Cx36_) responded to benzaldehyde presentation with a calcium decrease in both AWC and AIY, suggesting that ectopic gap junctions between AWC and AIY reversed the sign of the AWC–AIY connection from negative to positive (Fig. 2d, red). A similar effect was observed when the AWC_Cx36_AIY_Cx36_ transgenes were carried on a chromosomally integrated array (Supplementary Fig. 4). We also observed an attenuation in the degree of AWC hyperpolarization in the AWC_Cx36_AIY_Cx36_ worms, consistent with a shunting of the hyperpolarizing current to AIY (Fig. 2d, red). Together these results indicate that ectopic gap junctions were formed between AWC and AIY, switching an inhibitory chemical synapse to a predomininantly excitatory electrical/chemical synapse.

We also analysed the effect of ectopic connexin expression on responses to benzaldehyde removal. As expected, in wild-type worms this led to a calcium increase in AWC and a small calcium decrease in AIY, consistent with an inhibitory chemical synapse (Fig. 2e). In contrast, AWC_Cx36_AIY_Cx36_ worms showed a calcium increase in AIY, an attenuated calcium increase in AWC (Fig. 2e, red) and a strong defect in benzaldehyde chemotaxis (Supplementary Fig. 2b), consistent with the presence of an ectopic electrical connection. We also assayed AWC_Cx36_ and AIY_Cx36_ strains individually. We observed that AIY_Cx36_ worms showed normal responses to benzaldehyde removal and normal benzaldehyde chemotaxis, whereas AWC_Cx36_ worms showed reduced calcium influx and a partial deficiency in chemotaxis (Fig. 2h,i; Supplementary Fig. 2b). Since the left and right AWCs, unlike most amphid neurons, are not normally linked by gap junctions, we reasoned that AWC_Cx36_ expression might alter the neurons’ electrical properties by electrically coupling the AWC pair. Consistent with this possibility, when we expressed Cx36 in only one of the two AWCs, (AWC^off^) using the *srsx-3* promoter^29^, we observed wild-type calcium responses and normal chemotaxis (Supplementary Fig. 5), consistent with the possibility that the attenuation in AWC_Cx36_ activity was due to the formation of a new electrical connection between AWCR and AWCL. Alternatively, although our *odr-1* promoter fragment appears AWC-specific in our lines, if the *odr-1::Cx36* transgene is driving low-level Cx36 expression in other neurons, these might form ectopic gap junctions with the AWCs and thereby attenuate AWC responses by shunting.

## Discussion

In summary, we have shown using two different neural circuits in *C. elegans* that vertebrate connexins can be used to create synthetic electrical synapses in an intact nervous system. By transgenically targeting connexin expression to physically adjacent neurons, this manipulation can be used as a general technique for artificially modifying neural connectivity. This approach complements several existing methods for manipulating the excitability^30^ or efficacy of synaptic transmission^31^,^32^ from genetically targeted neurons. Ectopic connexin expression makes it possible not only to generate new connections between unconnected neurons, but also to modify the strength of existing electrical synapses^33^. Thus, synthetic modification of neural connections should prove to be a useful tool to obtain insight into the workings of neural circuits in genetically tractable animals, in which the precise connectivity and the relative position of each circuit component is known.

The approach described here takes advantage of the fact that vertebrate and invertebrate gap junctions are formed from distinct, most likely incompatible protein families. Thus, an ectoptic connexin hemichannel is not expected to pair with endogenous innexin hemichannels in *C. elegans* neurons; indeed, the observation that AWC_Cx36_ does not on its own lead to electrical coupling to AIY (which express the endogenous innexin *inx-18* (ref. 34)) supports this conjecture. Although this would appear to limit the exact method described here to invertebrate nervous systems, it has been found that invertebrate innexins can readily be expressed to form hemichannels in vertebrate *Xenopus* oocytes^14^. It is therefore feasible that a mirror image of the method we describe here (that is, ectopic expression of innexins in vertebrates) might be used to modify the connectivity of mammalian brain circuits. Synthetic synaptic modification approaches of this sort have a broad range of potential applications in neuroscience research, including the implementation of theoretical neural circuit models in real live circuits, the ability to test the sufficiency (as opposed to necessity) of particular connections for neuronal circuit function and even the engineering of novel circuits and behaviours.

## Methods

### Strains

Strains were grown and maintained under standard conditions. Wild-type animals were *C. elegans* Bristol strain N2. Other strains generated in this study were:

**AQ2660** ljEx351[odr-1::YC3.60*, elt-2::mCherry]; ljEx355[odr-1::Cx36*::mCherry, unc-122::mCherry].

**AQ2632** ljEx351[odr-1::YC3.60*, elt-2::mCherry].

**AQ2640** ljEx355[odr-1::Cx36*::mCherry, unc-122::mCherry].

**AQ2795** ljEx411[srsx-3::Cx36*::mCherry, unc-122::mCherry].

**AQ2817** ljEx413[srsx-3::YC3.60*].

**AQ2818** ljEx413[srsx-3::YC3.60*]; ljEx411[srsx-3::Cx36*::mCherry, unc-122::mCherry].

**AQ2610** ljEx336[ttx-3(int2)::Cx36*::mCherry, unc-122::gfp].

**AQ2614** ljEx339[odr-1::Cx36*::mCherry,ttx-3(int2)::Cx36*::mCherry, unc-122::mCherry].

**AQ2637** ljEx354[ttx-3(int2)::YC3.60*].

**AQ2646** ljEx354[ttx-3(int2)::YC3.60*]; ljEx339[odr-1::Cx36*::mCherry, ttx-3(int2)::Cx36*::mCherry, unc-122::mCherry].

**AQ2941** ljEx135[flp-6::YC2.12]; ljEx472[gcy-5::Cx36*::mCherry, gcy-7::Cx36*::mCherry, unc-122::mCherry].

**AQ3239** ljEx135[flp-6::YC2.12]; ljEx610[gcy-5::Cx36*::mCherry, unc-122::mCherry].

**AQ3309** *ljEx668[gcy-7::gfp, gcy-5::mCherry, unc-122::mCherry]*.

**AQ3310** ljEx669[gcy-7::Cx36*::yfp, gcy-5::Cx36*::mCherry, unc-122::mCherry].

**AQ3311** ljEx670[gcy-7::cx36*::mCherry, gcy-7::YC3.60, unc-122::mCherry].

**MF302** mfIs13[odr-1::Cx36*::yfp, ttx-3(int2)::Cx36*::mCherry, punc-122::mCherry].

### Visualizing Cx36 expression in transgenic animals

The complementary DNA sequence of *Mus musculus* gap junction protein, delta 2 (Gjd2) was codon optimized^23^ to produce a synthetic *Cx36* gene indicated in the strain list as Cx36* (GeneArt). We fused to Cx36 an upstream promoter (*gcy-5* for ASER^35^, *gcy-7* for ASEL^35^, *odr-1* for AWC^25^, *srsx-3* for AWC^off^^29^ and the second intron of *ttx-3* for AIY^36^) and a downstream gene encoding a fluorescent protein (YFP or mCherry). Plasmids were injected at 70 ng μl^−1^ each, together with a coinjection marker (*unc-122::gfp*, *unc-122::mCherry* or *elt-2::mCherry* at 40, 40 or 20 ng μl^−1^, respectively) to generate transgenic worms carrying an extrachromosomal array. To express an electrical synapse linking two neurons, we co-injected plasmids for both neurons in the same worm. Transgenic Cx36 expression was visualized using an LSM 510 or LSM 780 confocal laser scanning microscope (Zeiss), captured and processed by Zen (Zeiss) software and further processed using Volocity, Fiji and Adobe Illustrator CS3. After scanning a stack of ~1-μm-thick slices, either a maximum projection image or a three-dimensional rendering was generated and uniformly adjusted for brightness and contrast. Strains for imaging the overlap between the ASE neurons were prepared by co-injecting *gcy-7::gfp* and *gcy-5::mCherry* at 85 ng μl^−1^ each, with 40 ng μl^−1^ of *unc-122::mCherry.* These images were acquired and processed as mentioned above. For the deconvolution images in Supplementary Fig. 1, the samples were prepared by immobilizing the worms on a 3% pad of agarose using 200 mM of sodium azide. The worms were imaged using API Deltavision with a 100 × objective and processed using PerkinElmer Volocity.

### Chemotaxis assays

Salt chemotaxis assays were performed essentially as described^37^. Briefly, we poured 10 ml of buffered agar (2% agar, 1 mM CaCl2, 1 mM MgSO4, 5 mM KPO4) into 10-cm diameter Petri dishes. To form the gradient, 16 h before the assay we applied 10 μl of 250 mM NaCl salt solution adjusted to pH 6.0 to the attractant spot and 10 μl of double-distilled H_2_O to the control spot. Four hours before the assay, we added another 4 μl of salt solution or double-distilled H_2_O to the same spots. Ten minutes before the assay, we applied 1 μl of 1 M sodium azide to both attractant and control spots to immobilize the worms once they reached these spots. We used synchronized animals that were grown on 6-cm nematode growth media plates with food, washed three times with CTX solution (1 mM CaCl2, 1 mM MgSO4, 5 mM KPO4) and pipetted onto a nylon membrane to dry. Subsequently ~200 worms were placed in the center of the assay plate inside a circle of diameter of 1.5 cm. We allowed the animals to move about the agar surface for 1 h. The chemotaxis index was calculated as the number of animals contained in a circle of radius 1.5 cm around the salt spot minus the number of worms in a circle of radius 1.5 cm around the control spot, divided by the total number of animals in the two circles.

Benzaldehyde chemotaxis assays were performed as described^38^, except that they were performed in smaller 6-cm plates, and with *N*=20 worms, which were picked and transferred to the assay plate after being released from food on an empty plate. A drop of 1% benzaldehyde in ethanol and ethanol alone were placed at two opposite ends of the assay plate together with 1 mM sodium azide for trapping the worms. Chemotaxis was scored the following day by counting the number of paralyzed worms near the odour spot *N*(odour) and the number of paralyzed worms near the control spot *N*(control), and calculating the chemotaxis index^38^ equal to: (*N*(odour)−*N*(control))/(*N*(odour)+*N*(control)).

### Calcium imaging

Imaging was performed on a Zeiss Axioscope upright microscope using an Andor iXon EM camera or a Hamamatsu ORCA-ER camera in combination with neutral density filters to reduce the intensity of ultraviolet illumination. Movies were captured by IQ1.9 software (Andor) or a custom-written Matlab program and analysed using another custom-written Matlab (Mathworks) script. A rectangular region of interest (ROI) was drawn surrounding the cell body (AWC and ASE) or the neurite (AIY), and for every frame the ROI was shifted according to the new position of the center of mass (in the case of the cell body) or point of maximum intensity (for the AIY neural processes). The fluorescence intensity, *F*, was computed as the difference between the sum of pixel intensities and the faintest 10% pixels (background) within the ROI. For ratiometric imaging, ROI_Y_ tracked the neuron in the yellow channel, and in the cyan channel, ROI_C_ moved at a fixed offset from ROI_Y_. *F* was computed as *F*_Y_/*F*_C_ after correcting for bleed through^37^. No correction for bleaching was necessary. Δ*F* for calcium traces was equal to (*F*−*F*_0_)/*F*_0_ × 100, where *F*_0_ equals the average *F* within the first 3 s of recording. For statistical quantification Δ*F* was computed as (*F*_1_−*F*_0_)/*F*_0_ × 100, where *F*_0_ is the average *F* over 10 s before odour switching and *F*_1_ is the average over 10 s after odour switching. All imaging strains performed normally in chemotaxis assays. A two-tailed unpaired *t*-test was used to analyse the data. Where >1 comparison was made, a Bonferroni *t*-test was used instead.

To image salt responses, single animals were placed in a perfusion chamber (RC-26GLP,Warner Instruments) under a constant flow rate (0.3 ml min^−1^) of CTX buffer using a perfusion pencil (AutoMate). Outflow was regulated using a peristaltic pump (Econo Pump, Bio-Rad). Upsteps or downsteps in NaCl concentration were delivered using the perfusion pencil, and switch between control and stimulus solutions was done using manually controlled valves. Solutions contained the indicated amount of NaCl and in addition 1 mM MgSO_4_, 1 mM CaCl_2_ and 5 mM KPO_4_. The stimulus was delivered for 10 s starting on the 10th second from the onset of the movie.

To image olfactory responses, worms were inserted into a microfluidic polydimethylsiloxane chip, a generous gift from N. Chronis, designed to deliver odour stimuli under a fluorescent microscope^39^. Ten seconds after the beginning of each recording, we presented the odour (10^−4^ benzaldehyde diluted in S-basal lacking cholesterol) and continued imaging for an additional 30 s. We kept the odour channel open for 5 min and then resumed imaging. After an additional 10 s, we switched the odour off and completed the recording following an additional 30 s. We used buffer instead of fluorescein in the chip to enhance the signal to noise ratio and eliminate switching artifacts, and unlike previous reports^25^,^26^ we did not pre-immerse the worms in buffer before each recording.

## Additional information

**How to cite this article:** Rabinowitch, I. *et al.* Rewiring neural circuits by the insertion of ectopic electrical synapses in transgenic *C. elegans*. *Nat. Commun.* 5:4442 doi: 10.1038/ncomms5442 (2014).

## Supplementary Material

Supplementary Information Supplementary Figures 1-5
