# Supplementary material for: Rewiring neural circuits by the insertion of ectopic electrical synapses in transgenic C. elegans
Source: Nat Commun. 2014 Jul 16;5:4442. doi: 10.1038/ncomms5442 (PMC4109004; doi:10.1038/ncomms5442)
Supplement: Supplementary Information — Supplementary Figures 1-5 [file ncomms5442-s1.pdf]

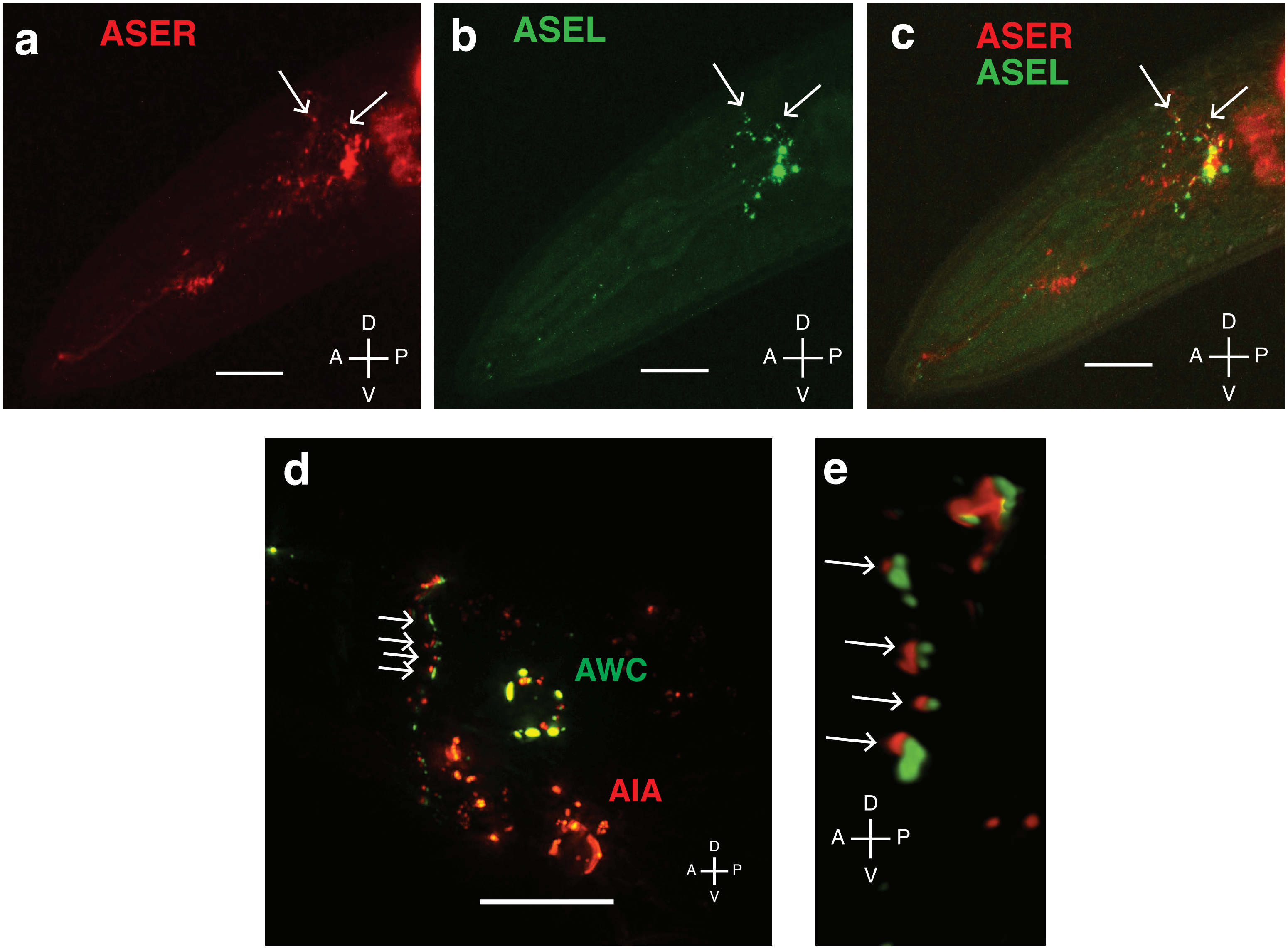

Supplemental Figure 1: Colocalisation of some Cx36 puncta in the nerve ring. Shown in panels a-c are confocal projection images of ASEL (green) and ASER (red) in an AQ3310 (see strain list) animal expressing YFP-tagged Cx36 in ASEL and mCherry-tagged Cx36 in ASER (strain AQ3310, see strain list). ASER::Cx36 (a) and ASEL::Cx36 (b) puncta are shown separately and in a merged image (c). Colocalizing puncta in the nerve ring are indicated with arrows. Scale bars are 20 μm. Panels d and e show deconvolved images of AWC (green) and AIA (red) in an AWC_{Cx36}; AIA_{Cx36} worm (AQ2644; *ljEx337[odr-1::Cx36*::yfp, unc-122::mCherry]; ljEx358[gcy-28.d::Cx36*::mCherry, unc-122:gfp]*). AWC_{Cx36} normally makes inhibitory chemical synapses with AIA_{Cx36} . (d) Maximum projection image showing AWC and AIA cell bodies (labeled) and adjacent puncta in the nerve ring (white arrows); scale bar is 10 μm. (e) Enlarged 3D rendering of the nerve ring; arrows indicate the same coincident puncta as panel d.

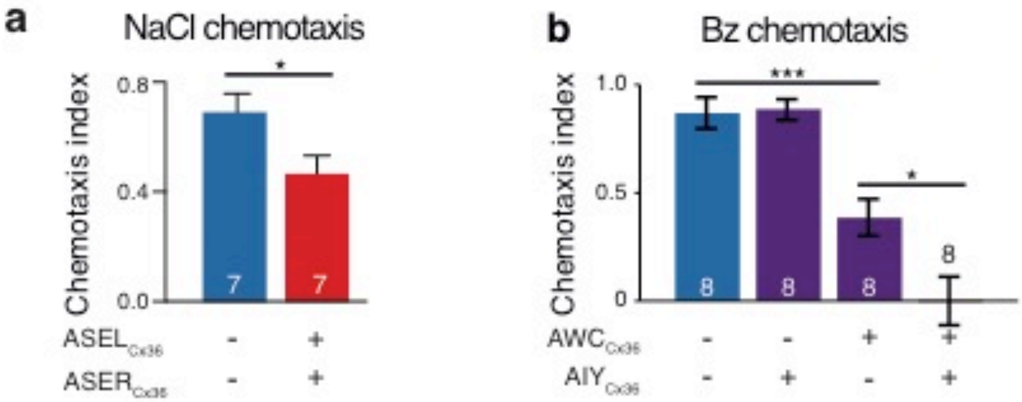

Supplementary Figure 2: Chemotaxis behavior of wild type and Cx36-expressing worms. Shown are chemotaxis scores for chemotaxis to NaCl (a) or benzaldehyde (b). Two-tailed unpaired t-tests with Bonferroni correction for multiple comparisons where relevant, *P<0.05, *** P<0.001, ns - not significant. Error bars represent SEM. Numbers on bars indicate the number of trials (200 animals/experiment for NaCl; 20 animals/experiment for benzaldehyde).

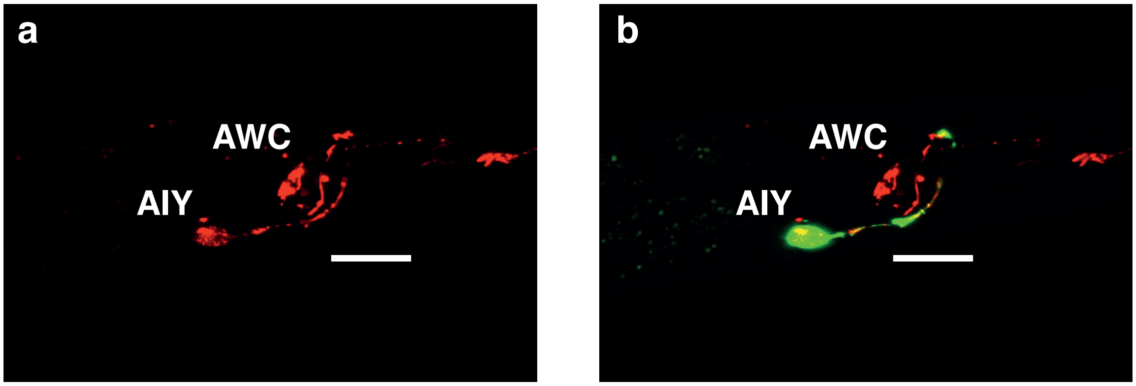

Supplementary Figure 3: Expression of Cx36 in AWC and AIY. Shown are confocal projection images of transgenic line AQ2646, in which fluorescently-tagged Cx36 is expressed in AIY and AWC (red punctate fluorescence, panels a and b), and the genetically-encoded calcium indicator YC3.60 is expressed in AIY (green fluorescence in merged image, panel b). Scale bar, 10 µm.

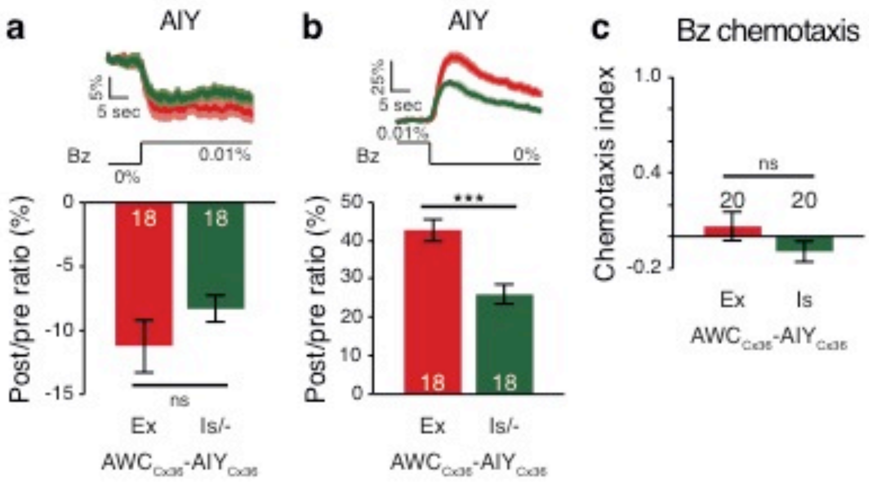

Supplementary Figure 4: Genomic integration of Cx36. (**a**,**b**) Calcium imaging of AIY responses to an up-step (a) or down-step (b) in benzaldehyde (Bz) concentration in worms harboring a Cx36 extrachromosomal or genomically integrated transgene in both AWC and AIY. Since we were unable to obtain homozygotic cross-progeny of the integrated $AWC_{Cx36}AIY_{Cx36}$ line and an AIY cameleon line, we used heterozygotes obtained by crossing strains AQ2637 and MF302. (**c**) Chemotaxis scores of extrachromosomal or integrated $AWC_{Cx36}AIY_{Cx36}$ worms. Two-tailed unpaired t-tests with Bonferroni correction for multiple comparisons where relevant, *P<0.05, ns - not significant. Error bars represent SEM. Numbers on bars indicate sample sizes.

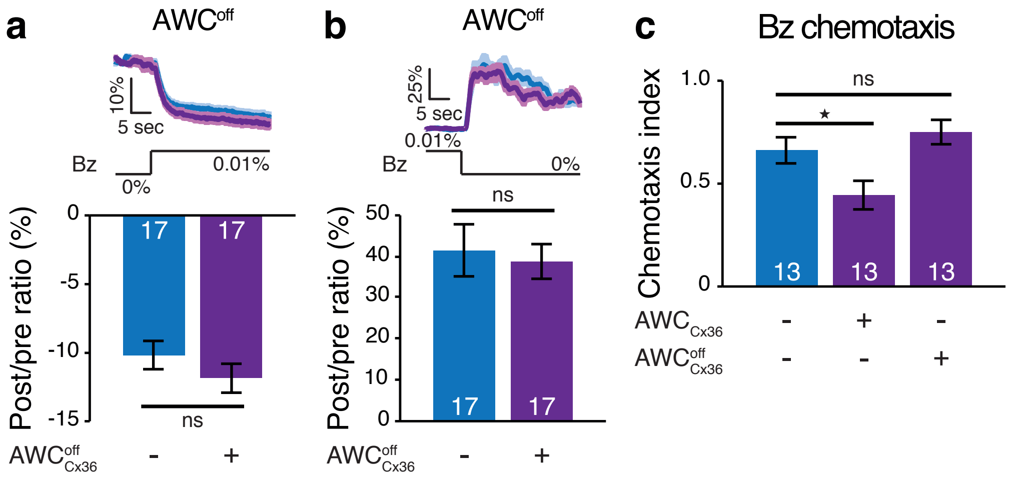

Supplementary Figure 5: Cx36 expression in AWC^{off} alone. (**a**,**b**) Calcium imaging of AWC^{off} responses to an up-step (a) or down-step (b) in benzaldehyde (Bz) concentration in wild type worms and in worms expressing Cx36 in AWC^{off}. The *srsx-3* promoter drives Cx36 expression as expected in the two AWB neurons and one AWC (i.e. AWC^{off}). (**c**) Chemotaxis scores of wild type and Cx36-expressing worms. Two-tailed unpaired t-tests with Bonferroni correction for multiple comparisons where relevant, *P<0.05, ns - not significant. Error bars represent SEM. Numbers on bars indicate sample sizes.
